# Elovl2 ablation demonstrates that systemic DHA is endogenously produced and is essential for lipid homeostasis in mice[S]

**DOI:** 10.1194/jlr.M046151

**Published:** 2014-04

**Authors:** Anna M. Pauter, Petter Olsson, Abolfazl Asadi, Bengt Herslöf, Robert I. Csikasz, Damir Zadravec, Anders Jacobsson

**Affiliations:** * Department of Molecular Biosciences, The Wenner-Gren Institute, Stockholm University, SE-10691 Stockholm, Sweden; and; † Department of Analytical Chemistry, Stockholm University, SE-10691 Stockholm, Sweden

**Keywords:** fatty acid elongase, elongase of very long-chain fatty acid 2, docosahexaenoic acid, liver steatosis, weight gain

## Abstract

The potential role of endogenously synthesized PUFAs is a highly overlooked area. Elongation of very long-chain fatty acids (ELOVLs) in mammals is catalyzed by the ELOVL enzymes to which the PUFA elongase ELOVL2 belongs. To determine its in vivo function, we have investigated how ablation of ELOVL2, which is highly expressed in liver, affects hepatic lipid composition and function in mice. The *Elovl2^−/−^* mice displayed substantially decreased levels of 22:6(n-3), DHA, and 22:5(n-6), docosapentaenoic acid (DPA) n-6, and an accumulation of 22:5(n-3) and 22:4(n-6) in both liver and serum, showing that ELOVL2 primarily controls the elongation process of PUFAs with 22 carbons to produce 24-carbon precursors for DHA and DPAn-6 formation in vivo. The impaired PUFA levels positively influenced hepatic levels of the key lipogenic transcriptional regulator sterol-regulatory element binding protein 1c (SREBP-1c), as well as its downstream target genes. Surprisingly, the *Elovl2^−/−^* mice were resistant to hepatic steatosis and diet-induced weight gain, implying that hepatic DHA synthesis via ELOVL2, in addition to controlling de novo lipogenesis, also regulates lipid storage and fat mass expansion in an SREBP-1c-independent fashion. The changes in fatty acid metabolism were reversed by dietary supplementation with DHA.

As a source of energy and as structural components of membranes, omega-3 (n-3) and omega-6 (n-6) PUFAs have been shown to have significant roles in many biological functions, including immunity, growth, and metabolism in mammals. Dietary PUFAs, and especially DHA, have been shown to influence hepatic liver metabolism and prevent the development of liver steatosis (1–4). Nevertheless, the role of endogenously synthesized PUFAs compared with PUFAs taken up from the diet is relatively unknown.

In mammals, the synthesis of very long-chain PUFAs requires a dietary supply of the essential precursors of the omega-6 and omega-3 series, linoleic acid, C18:2n-6, and α-linolenic acid, C18:3n-3, respectively (5). The biosynthesis of PUFAs of the n-6 and n-3 series occurs via sequential desaturation and elongation steps by the Δ5- and Δ6-fatty acid desaturases (FADSs) 1 and 2, and the fatty acid elongation of very long-chain fatty acids (ELOVLs) 5, 2, and 4 (6). However, the biosynthesis of the omega-6 PUFA C22:5n-6, docosapentaenoic acid (DPA) n-6, and the omega-3 PUFA C22:6n-3, DHA, requires 24-carbon intermediates, which are subsequently desaturated prior to chain shortening through partial β-oxidation, ultimately yielding DPA and DHA (7, 8).

ELOVL5 has been proposed to be involved in the elongation of 18- and 20-carbon PUFA substrates, while data indicate that ELOVL2 and ELOVL4 elongate C20–C24 and C26–C34 PUFAs, respectively (9–12).

While ELOVL5, FADS1, and FADS2 are expressed to a certain extent in all tissues tested, the level of ELOVL2 is significant in liver, testis, uterus, placenta, mammary gland, retina, and certain areas of the brain, all of which are tissues that are documented as being rich in DHA (13, 14). In contrast to testis and retina, PUFAs longer than C24 are almost undetectable in liver, mainly due to the low level of *Elovl4* expression in this tissue, while PUFAs up to C22 are quite abundant within both the phospholipid and TG pools of hepatocytes (15). As the first enzyme in the PUFA elongation chain, ELOVL5 activity is suggested to affect multiple pathways regulating hepatic lipid and carbohydrate composition in mammals (11), while the role for ELOVL2 in liver is unknown.

Our previous study, concerning the role of ELOVL2 in sperm maturation and male fertility, revealed that *Elovl2^−/−^* mice are infertile and heterozygote *Elovl2^+/−^* C57Bl/6 mice exhibit haploinsufficiency that gives rise to impaired spermatides in >99% of all male mice, which hindered us from obtaining homozygous *Elovl2^−/−^* mice (16). To overcome this, we backcrossed *Elovl2^+/−^* females with 129S2/Sv males enabling the production of fertile *Elovl2^+/−^* males and subsequently generation of *Elovl2^−/−^* mice.

In vitro data have suggested a role of ELOVL2 in the elongation of C20–C24 PUFAs. By using ELOVL2-ablated mice, we now show that the major in vivo substrates of ELOVL2 are C22:5n-3 and C22:4n-6. As a consequence, ELOVL2 completes the final elongation step in the synthesis of C22:5n-6 and C22:6n-3 in liver.

Our data also reveal, for the first time, the physiological consequences of impaired ELOVL2 activity and endogenous DHA synthesis. The *Elovl2*-ablated mice have significantly distorted levels of C22 PUFAs in serum that correlate extremely well with the levels seen in both the phospholipid and TG pools in liver. Particularly, the mutant mice show a massive (20-fold) reduction in the ratio of the omega-3 PUFAs 22:6n-3 and 22:5n-3, implying hepatic ELOVL2 as a dominating factor in the control of serum levels of DHA. As a consequence, despite an induced nuclear form of hepatic sterol-regulatory element binding protein 1c (SREBP-1c), the *Elovl2^−/−^* mice show a reduced capacity to accumulate fat, which is reversible by dietary DHA supplementation.

## MATERIALS AND METHODS

### Animals

*Elovl2^−/−^* mice were generated as described previously (16) and backcrossed 129S2/Sv for four generations. All animals were housed at room temperature and maintained on a 12 h light/dark cycle. Ten- to 17-week-old male mice were single caged and fed standard chow diet for 12 weeks (<10% kcal fat, Labfor R70; Lantmännen, Sweden) or a high-fat diet (45% kcal fat, D12451; Research Diets, New Brunswick, NJ). In addition, 17- to 18-week-old male mice were fed standard chow diet or DHA-enriched chow diet for 2 weeks (Quimper, France) followed by 2 weeks of high-fat diet treatment. For the fatty acid composition of diets, see supplementary Table II. All animals were fed ad libitum and had free access to water. As control mice, age-matched littermates from a heterozygote breeding were used and housed under the same conditions as the *Elovl2^−/−^* mice. Body weight and food consumption were measured weekly as indicated in the Results section. At the end of the study, animals were euthanized with CO_2_. Relevant tissues were frozen in liquid nitrogen and stored at −80°C until required. All studies were carried out with ethical permission from the Animal Ethics Committee of the North Stockholm region, Sweden.

### Lipid extraction

Liver extracts were prepared by grinding a small piece of frozen sample to a fine powder using a ceramic mortar in the presence of liquid nitrogen. Fifty milligrams was transferred to a glass test tube with a teflon-lined screw cap. Twenty microliters of triheptadecanoin (10.0 mg/ml in chloroform) and 40 µl of 1,2-diheptadecanoyl-sn-glycero-3-phosphatidylcholine (5.0 mg/ml in chloroform) were added, and the sample was dried in a stream of nitrogen. The extraction protocol then followed the procedure earlier published by Matyash et al. (17). Prior to HPLC separation, the samples were redissolved in chloroform-methanol (1:2, v/v). Serum extracts were obtained by centrifugation of fresh blood samples at 1,800 *g* for 20 min according to Matthan et al. (18) and were then stored in −80°C until use. Fifty microliters was transferred to a glass test tube with a teflon-lined screw cap. Twenty microliters of triheptadecanoin (10.0 mg/ml in chloroform) was added, the sample was dried in a stream of nitrogen, and lipids were extracted according to Matyash et al. (17). All solvents were of HPLC grade and purchased from Rathburn Chemicals Ltd. (Walkerburn, Scotland). Lipid standards were purchased from Larodan Fine Chemicals AB (Malmö, Sweden) with purity ≥99%. Boron trifluoride (BF_3_, 14% in methanol) was purchased from Sigma Aldrich (Milwaukee, WI).

### Lipid fractionation and analysis

Fractionation of liver samples was performed using preparative HPLC by modification of an earlier published method (19). An HP-1050 quaternary pump (Agilent Technologies Inc., Santa Clara, CA) was fitted to a Reprosil-Pur 120 CN column [250 mm × 10 mm inner diameter (ID), 5 µm particle size; Dr. Maisch GmbH, Ammerbuch, Germany] and a Reprosil-Pur CN guard column (30 mm × 10 mm ID, 5 µm particle size; Dr. Maisch GmbH). An evaporative light-scattering detector (DDL21; Cunow, St. Christophe, France) was used for detection. Elution was performed using a binary mixture consisting of mobile phase A (heptane) and mobile phase B [toluene-methanol-acetic acid-triethylamine (60:40:0.2:0.1, by weight)]. A gradient was applied after 3 min by increasing B from 10% to 40% in 7 min. Then B was further increased to 50% before being decreased again to 10% in 1 min. The total mobile flow rate was set to 4.7 ml/min and split 1:10 using a t-connection, where the lower-flow end was connected to the detector and the high-flow end was used for fraction collection. Eighty microliters of mouse liver extract was injected, and the neutral lipid fraction was collected from 2 to 5 min, and the polar lipid fraction from 11 to 14 min into glass test tubes provided with teflon-lined screw caps. After collection, all samples were dried in a stream of nitrogen and then subjected to transesterification and analyzed by GC as described previously (20).

### MRI measurement

In vivo MRI using EchoMRI-100^TM^ (Echo Medical Systems, Houston, TX) was performed in order to measure body fat and lean content.

### Indirect calorimetry

To determine resting metabolic rate, oxygen consumption was measured by indirect calorimetry (INCA systems; Somedic, Hörby, Sweden) as previously described (21). Mice were subjected to oxygen consumption measurements at room temperature for 22 h, starting at 0800. Animals were fed ad libitum and had free access to water. Animals in their home cages were put into sealed chambers (vol 4 liters). The air leaving the chamber was dried with silica gel (Safegel 1–3 mm with yellow moisture indicator, Merck Prolabo; VMR International, West Chester, PA). The zirconium oxide sensors were calibrated daily using two reference gases containing 18% and 25% oxygen in nitrogen before measurement. Measurements proceeded under a constant airflow rate (1liter/min). Oxygen consumption (VO_2_) and carbon dioxide production (VCO_2_) were recorded every 2 min. Resting energy expenditure (REE) was calculated according to the Weir formula (REE = 16.3 VO_2_ + 4.57 VCO_2_) and normalized to the lean weight per animal.

### Histology

Livers were fixed in 10% neutral buffered formalin, sectioned, stained with hematoxylin and eosin, and examined by light microscopy histological evaluation.

### Quantitative RT-PCR

RNA was isolated with TRI Reagent (Sigma Aldrich), and total RNA was isolated following the manufacturer's procedure. For real-time PCR, 500 ng total RNA was reverse transcribed using random hexamer primers, deoxynucleoside triphosphates, MultiScribe reverse transcriptase, and RNase inhibitor (Applied Biosystems, Foster City, CA). cDNA samples were diluted 1:10, and aliquots of 2 μl of the sample cDNA were mixed with SYBR Green JumpStart Taq ReadyMix (Sigma Aldrich), prevalidated primers, and diethylpyrocarbonate-treated water and were measured in duplicate for each sample. Primers used were fatty acid synthase (FAS), SREBP-1c, sterol-CoA desaturase 1 (SCD1), PPARγ, and phosphoenolpyruvate carboxykinase 1 (Pck1). For sequences, see supplementary Table VIII. Expression analysis was performed using the BioRad detection system. Data were normalized to the housekeeping gene 18S.

### Nucleic protein extraction

Five hundred milligrams of frozen liver was placed in ice-cold PBS and subsequently homogenized in 10 mM HEPES, pH 7.6, 25 mM KCl, 0.15 mM spermine, 0.5 mM spermidine, 1 mM EDTA, 2 mM sucrose, 10% glycerol, 2 mM DTT, 0.1 mM PMSF, and one protease inhibitor tablet (Complete Protease Inhibitor tablets; Roche) per 10 ml and subjected to centrifugation at 20,000 rpm for 1 h at 4°C in a JA20 rotor. The pellet was resuspended in 10 mM HEPES, pH 7.6, 100 mM KCl, 0.1 mM EDTA, 3 mM MgCl_2_, 10% glycerol, 1 mM DTT, 0.1 mM PMSF, and one protease inhibitor tablet (Complete Protease Inhibitor tablets; Roche) per 10 ml, and 1/10 vol of 4 M (NH_4_)_2_SO_4_ was added. After 40 min of incubation on ice, the solution was subjected to ultracentrifugation at 85,000 rpm for 45 min at 4°C in a TLV 100K rotor. The supernatant was collected as a nuclear protein extract. Protein concentration was determined using the Lowry Assay.

### Western blotting

For immunoblotting of SREBP-1c, the nuclear protein extracts from each sample (10 μg/lane) were separated by 10% SDS-PAGE and blotted to a polyvinylidene difluoride transfer membrane (Amersham Hybond-P; GE Healthcare) in a semidry system. The membrane was incubated for 1 h in 5% fat-free milk, then overnight with the primary SREBP-1c antibody (2A4: sc-13551; Santa Cruz Biotechnology) diluted 1:1,000 in 5% BSA, 1× TBS, and Tween-20 and for 1 h with horseradish peroxidase-conjugated secondary antibody (anti-mouse; Cell Signaling) diluted 1:2,000 in 5% fat-free milk, 10× TBS, and Tween-20. The membrane was washed with TBS and Tween-20 2 × 5 min and 15 min after each incubation time. Proteins were visualized using an ECL Plus kit (Amersham Bioscience) and detected in an LAS-1000 CCD camera (Fuji).

### Statistical analysis

Statistical analysis was performed using GraphPad PRISM (San Diego, CA), and statistical differences were calculated with Student's unpaired *t*-test.

## RESULTS

### ELOVL2 controls the level of the omega-3 fatty acid DHA in liver and serum

**Fig. 1.**
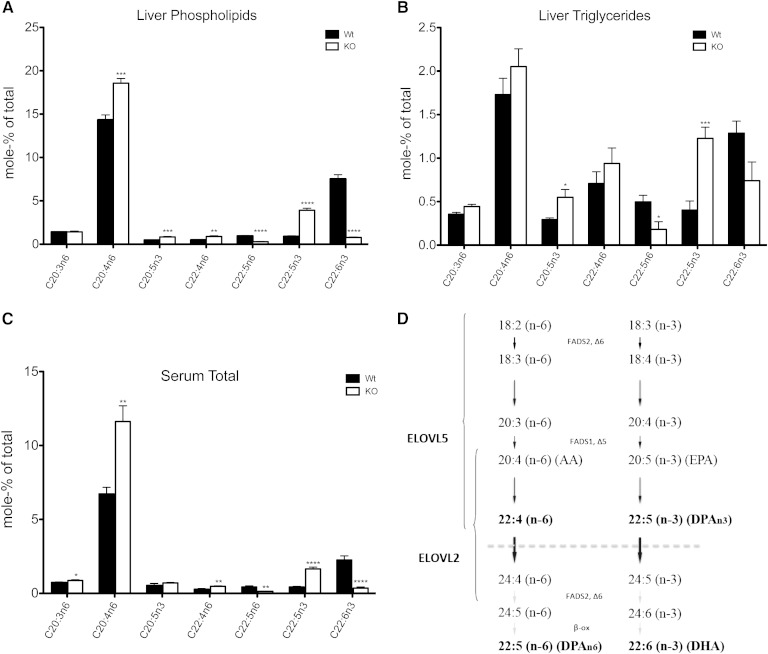
ELOVL2 is the sole elongase that elongates C22–C24 PUFA in liver. PUFA composition of phospholipid pool (A) and TG pool (B) from liver, as well as total fatty acid composition of serum (C) in wild-type and *Elovl2^−/−^* animals fed standard chow diet. For the % mole composition of the remaining fatty acids, see supplementary Tables III, IV, and V, respectively. Biosynthesis of PUFAs in the liver (D) where ELOVL2 specifically elongates 22:5n-3 and 22:4n-6 fatty acids to form precursors (24:5n-3 and 24:4n-6) for DHA and DPAn-6 formation. Results shown are means ± SEM of 3–6 mice. Statistical significances are shown between wild-type and *Elovl2^−/−^* mice (* *P* < 0.05, ** *P* < 0.01, *** *P* < 0.001, and **** *P* < 0.0001).

Considering the role of ELOVL2 and PUFA synthesis in liver, we analyzed by GC in combination with HPLC fractionation the hepatic fatty acid composition in both the phospholipids and TG pools in *Elovl2*-ablated mice and compared this with the fatty acid composition in serum. We observed an accumulation of the omega-6 fatty acids 20:4n-6 (arachidonic acid, AA) and 22:4n-6, as well as the omega-3 fatty acids 20:5 (EPA) and 22:5n-3, in both liver and serum of *Elovl* KO mice compared with wild-type littermates. This was accompanied by a massive (90%) decrease in the levels of 22:5n-6 and 22:6n-3, respectively (**Fig. 1A–C**). This clearly demonstrates a unique role of ELOVL2 in the elongation process of C22 into C24 PUFAs and, consequently, in the formation of DHA in liver (Fig. 1D). Moreover, the fatty acid profile in serum was reflected entirely by the hepatic lipid composition, indicating liver as the major contributor of serum DHA under standard dietary conditions.

### Deficiency in the endogenously synthesized DHA activates hepatic SREBP-1c and stimulates transcription of lipogenic genes

**Fig. 2.**
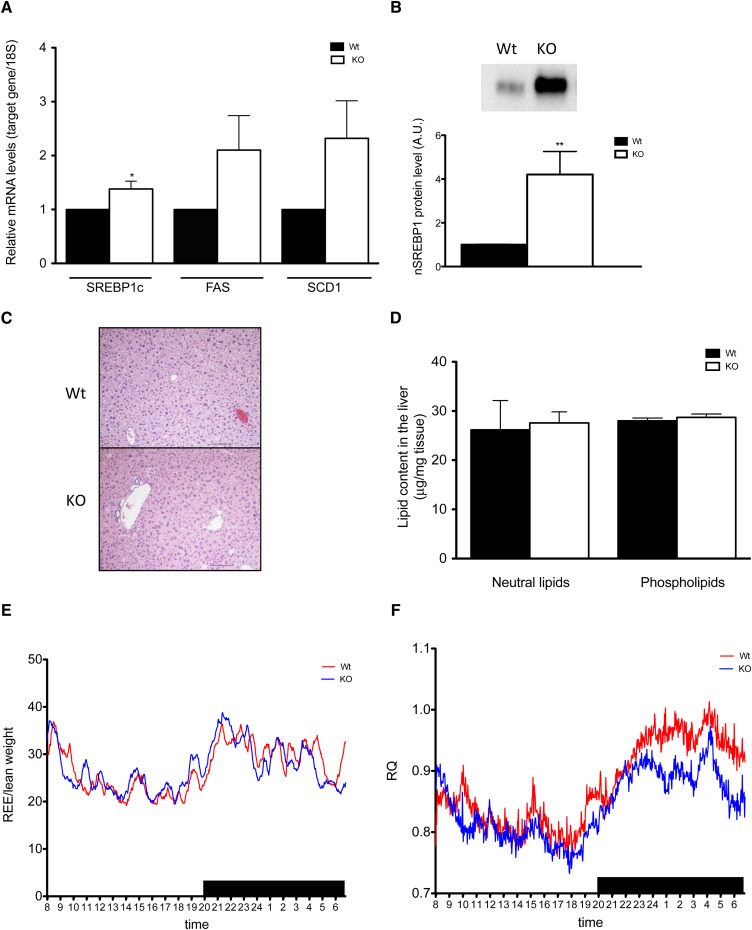
Upregulation of lipogenic gene expression does not result in hepatic lipid accumulation in *Elovl2^−/−^* mice fed standard chow diet. Analysis of hepatic gene expression of SREBP-1c, FAS, and SCD1 (A) in wild-type and *Elovl2^−/−^* mice fed standard chow diet. nSREBP-1c protein expression in hepatic nuclear extracts from wild-type and *Elovl2^−/−^* animals fed standard chow diet (B). Microscopic overview (C) of liver tissue from wild-type and *Elovl2^−/−^* mice fed standard chow diet and content of neutral lipids and phospholipids (D). Time course showing REE (E) and mean values of respiratory quotient (RQ) (F) during a 22 h period for wild-type and *Elovl2^−/−^* mice fed standard chow diet. Results shown are means ± SEM of 3–6 mice (exception E and F). Statistical significances are shown between wild-type and Elovl2^−/−^. * *P* < 0.05, ** *P* < 0.01, and *** *P* < 0.001.

It has been reported that one of the beneficial effects of PUFAs, especially DHA, on lipid metabolism is via their involvement in the regulation of the key lipogenic transcriptional factor SREBP-1c. As dietary supplementation with DHA has been shown to both repress hepatic transcription and to positively influence the degradation of the nuclear, active form of SREBP-1c (nSREBP-1c), DHA potentially drives deactivation of SREBP-1c target genes, such as FAS and SCD1, and suppress de novo lipogenesis in mice (22–24). In line with this, the *Elovl2*-ablated mice, which are deficient in DHA, showed significantly elevated hepatic mRNA levels of SREBP-1c (**Fig. 2A**), as well as nSREBP-1c protein (Fig. 2B). As expected, upregulation of SREBP-1c resulted in the activation of the downstream target genes for FAS and SCD1 (Fig. 2A). No differences were detected in the level of diacylglycerol acyltransferase 2, carnitine palmitoyltransferase 1, and acetyl-CoA carboxylase, indicating normal TG formation and fatty acid oxidation in the KO mice. These findings are in accordance with previous data showing a negative regulation of SREBP-1c by dietary DHA exposure, as well as with the study on *Elovl5*-ablated mice that reports an activation of SREBP-1c under conditions of reduced levels of endogenously synthesized PUFAs longer than C18 (25).

### SREBP-1c activation is not followed by TG accumulation in the liver of *Elovl2^−/−^* mice

Activation of SREBP-1c and its target genes has been proposed as a major factor in the development of hepatic steatosis due to stimulation of fatty acid and TG synthesis (26). Recently, Moon and coworkers (25) showed that *Elovl5* ablation and reduced levels of PUFA lead to steatosis by impaired suppression of SREBP-1c. Surprisingly, despite upregulation in hepatic lipogenic gene expression (Fig. 2A), *Elovl2^−/−^* mice did not show accumulation of liver TGs (Fig. 2D), and from the histological analysis, we can conclude that *Elovl2^−/−^* liver does not significantly differ from wild-type hepatic tissue and did not show any signs of exaggerated fat storage and steatosis (Fig. 2C).

Furthermore, there was no significant difference in body weight, lean weight, fat mass, and energy intake between *Elovl2^−/−^* mice and wild-type littermates (supplementary Table I). In addition, we did not observe any disparity regarding fasting blood glucose levels (7.18 and 7.68 mM between wild-type and Elovl2^−/−^ mice, respectively), TG and cholesterol levels, or response to glucose tolerance test between *Elovl2^−/−^* mice and wild-type littermates. From indirect calorimetric measurements, we could detect a tendency for the *Elovl2*-ablated animals to have higher energy expenditure (resting metabolic rate) than wild-type mice in the light period and at the beginning of the dark period (Fig. 2E), although this difference in energy expenditure was slightly reversed during the later stage of the dark phase. When we analyzed the calorimetric parameter RQ, we found that it was significantly lower during the dark period, when the mice are more active and have their major food intake, implying that the DHA-deficient animals more promptly utilized lipids for energy production than the wild-type mice (Fig. 2F). Taking these findings together, we can conclude that a higher lipid oxidation contributes to lower levels of fat accumulation in the *Elovl2*-ablated animals.

### High-fat diet affects lipid composition in the liver and serum of *Elovl2^−/−^* mice but does not lead to hepatic steatosis

**Fig. 3.**
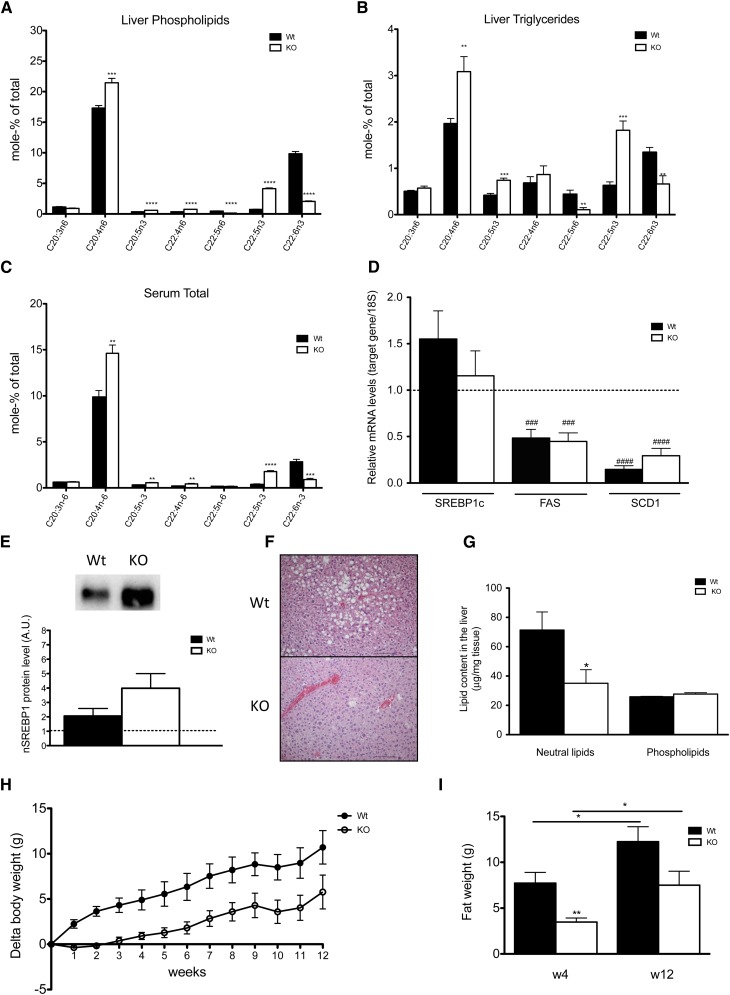
Resistance of *Elovl2^−/−^* mice to diet-induced liver steatosis. PUFA composition of phospholipid pool (A) and TG pool (B) from liver, and total fatty acid composition of serum (C) in wild-type and *Elovl2^−/−^* animals fed high-fat diet for 12 weeks. For the % mole composition of remaining fatty acids, see supplementary Tables III–V. Relative hepatic gene expression of SREBP-1c, FAS, and SCD1 (D) in wild-type and *Elovl2^−/−^* mice fed high-fat diet compared with values in wild-type mice fed chow diet (dotted line). Immunobloting of nSREBP-1c protein in hepatic nuclear extract in wild-type and *Elovl2^−/−^* animals fed high-fat diet compared with values in wild-type mice fed chow diet (dotted line) (E). Microscopic overview (F) of liver tissue from wild-type and *Elovl2^−/−^* fed high-fat diet for 12 weeks and of content of neutral lipids and phospholipids (G). Increase in body weight during 12 weeks of high-fat diet treatment presented as delta values for wild-type and *Elovl2^−/−^* mice (H). Fat content in wild-type and *Elovl2^−/−^* animals fed high-fat diet for 12 weeks measured at two time points: 4 weeks (W4) and 12 weeks (W12) (I). Results shown are means ± SEM of 3–6 mice. Statistical significances are shown between wild-type and Elovl2^−/−^ mice, as well as different time points (* *P* < 0.05, ** *P* < 0.01, *** *P* < 0.001, and **** *P* < 0.0001) and versus control values (mice fed chow diet) (# *P* < 0.05, ## *P* < 0.01 and ### *P* < 0.001, #### *P* < 0.0001).

High-fat diet is considered to be one of the main stressors that can induce negative effects in animals such as metabolic syndrome with obesity, insulin resistance, and fatty liver. In conjunction with this, it has been reported that a high-fat diet regime increases the level of hepatic lipogenesis and induces hepatic steatosis via upregulation of SREBP-1c (22, 27). To further investigate how DHA-deficient *Elovl2*-ablated animals, which already have induced hepatic levels of SREBP-1c, respond to this kind of stress condition, animals were given a high-fat (40%) diet treatment for 12 weeks. Although the consumption of high-fat diet affected the lipid composition in both liver and serum (supplementary Tables III–V), the relative differences in the PUFA profile between *Elovl2^−/−^* and control mice, seen under standard chow diet conditions (Fig. 1A–C), remained (**Fig. 3A–C**). In accordance with the expected findings, wild-type mice on high-fat diet compared with standard chow diet (set to 1) showed elevated levels of both SREBP-1c mRNA (Fig. 3D) and nSREBP-1c protein (Fig. 3E) in liver. Although the treatment with high-fat diet induced SREBP-1c mRNA levels even further in *Elovl2^−/−^* mice, there was no significant difference in absolute values between *Elovl2*^−/−^ and wild-type mice (Fig. 3D). However, *Elovl2*-ablated animals showed higher levels of nSREBP-1c protein after the treatment compared with wild-type littermates (Fig. 3E). Interestingly, and in contrast to the data from *Elovl2^−/−^* animals maintained on standard chow diet, upregulation of nSREBP-1c by high-fat diet did not result in increased, but rather in decreased, expression of the genes of lipogenic enzymes FAS and SCD1 (Fig. 3D) in both wild-type and *Elovl2^−/−^* mice, suggesting an SREBP-1c-independent mechanism in the control of lipogenesis under these conditions. Surprisingly, despite superinduced levels of nSREBP-1c by high-fat diet, *Elovl2^−/−^* animals did not show any signs of fatty liver (Fig. 3F), and the TG levels were significantly lower than in wild-type mice (Fig. 3G), which again accentuates the observation that reduced levels of DHA in these mice are protective against fatty liver.

### Resistance to diet-induced weight gain in *Elovl2^−/−^* mice was abolished upon dietary DHA supplementation

In accordance with reduced TG levels in liver, *Elovl2^−/−^* mice maintained on high-fat diet did not gain as much weight in comparison with their wild-type littermates (Fig. 3H). This difference was mainly due to lower fat mass in the *Elovl2^−/−^* mice measured at two different time points (Fig. 3I), where the first 4 weeks of high-fat diet treatment seemed to be the most dramatic period when the *Elovl2^−/−^* animals showed almost no increase in weight or fat gain in comparison with their wild-type littermates. No difference in food intake was observed between KO and control littermates (*Elovl2^−/−^* 389.5 ± 7.5 vs. wild type 384.6 ± 5.7 kJ/week). One possible explanation for the discrepancy in weight gain between short-term and long-term high-fat feeding of *Elovl2^−/−^* mice may be that trace amounts of DHA within the high-fat diet lead to a small but significant increase in DHA accumulation with time in these mice (supplementary Tables III–V).

**Fig. 4.**
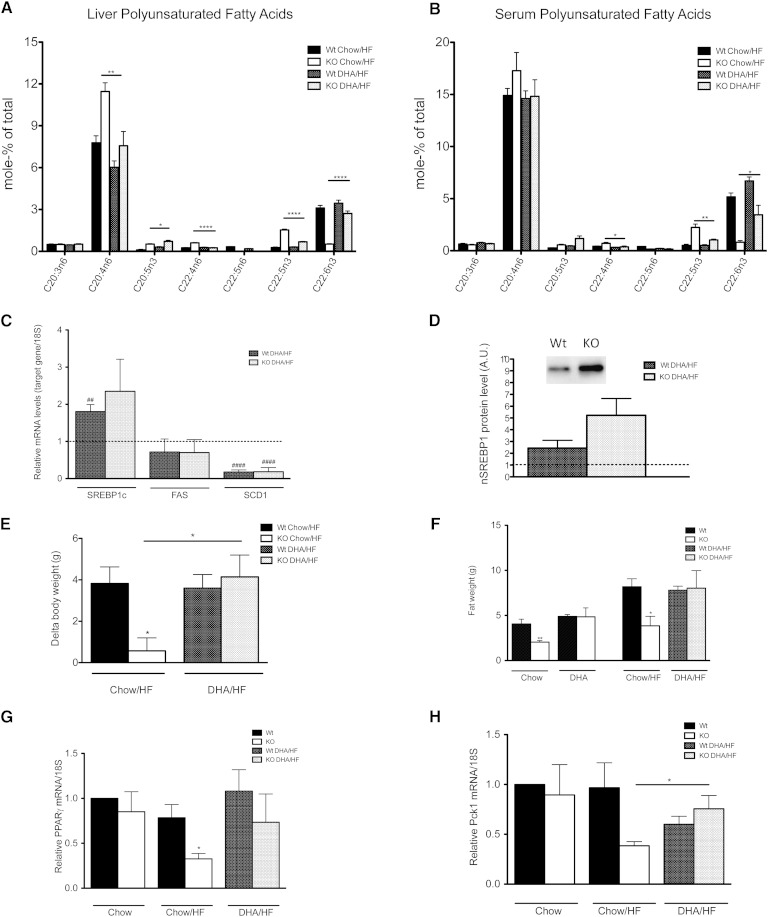
DHA supplementation abolished resistance of weight gain in *Elovl2^−/−^* mice. Total PUFA composition of liver (A) and serum (B) of wild-type and *Elovl2^−/−^* animals fed standard chow diet followed by 2 weeks of high-fat diet (Chow/HF) or prefed for 2 weeks with DHA-enriched diet followed by 2 weeks of high-fat diet (DHA/HF). For the experimental overview, see supplementary Fig. I, and for the % mole composition of remaining fatty acids, see supplementary Tables VI and VII. Relative hepatic gene expression of SREBP-1c, FAS, and SCD1 (C) in wild-type and *Elovl2^−/−^* mice fed for 2 weeks with DHA-enriched diet followed by 2 weeks of high-fat diet (DHA/HF) relative to values in wild-type mice fed chow diet (dotted line). Relative levels of nSREBP-1c protein in wild-type and *Elovl2^−/−^* animals fed for 2 weeks with DHA-enriched diet followed by 2 weeks of high-fat diet (DHA/HF) relative to values in wild-type mice fed chow diet (dotted line) (D). Increase in body weight of wild-type and *Elovl2^−/−^* animals fed standard chow diet followed by 2 weeks of high-fat diet (Chow/HF) or prefed for 2 weeks with DHA-enriched diet followed by 2 weeks of high-fat diet (DHA/HF), presented as mean of delta values after third week of treatment (E). Fat content in wild-type and *Elovl2^−/−^* animals measured at two time points: after 2 weeks prefed with standard chow diet (Chow) or DHA-enriched diet (DHA) followed by 2 weeks of high-fat diet (Chow/HF and DHA/HF), respectively (F). Relative hepatic gene expression of PPARγ (G) and Pck1 (H) in wild-type and *Elovl2^−/−^* mice fed standard chow diet (Chow), standard chow diet followed by 2 weeks of high-fat diet (Chow/HF), or 2 weeks with DHA-enriched diet followed by 2 weeks of high-fat diet (DHA/HF) relative to values in mice fed chow diet. Results shown are means ± SEM of 3–5 mice. Statistical significances are shown between wild-type and Elovl2^−/−^ mice, as well as different diets (* *P* < 0.05, ** *P* < 0.01, *** *P* < 0.001, and **** *P* < 0.0001) and versus control values (mice fed chow diet) (# *P* < 0.05, ## *P* < 0.01 and ### *P* < 0.001, #### *P* < 0.0001).

To determine whether dietary DHA may improve the weight gain and lipid accumulation of the *Elovl2^−/−^* mice, we provided a 2 week pretreatment with a DHA-supplemented diet prior to a 2 week high-fat exposure. As seen in **Fig. 4A, B**, the treatment resulted in a significant increase in DHA levels in both liver and serum, respectively, which concomitantly led to a reduced DPAn-3/DHA ratio in both wild-type and *Elovl2*^−/−^ mice. Surprisingly, despite normalized levels of DHA (supplementary Tables VI and VII), the hepatic level of nSREBP-1c protein still remained high (Fig. 4D), and the lipogenic gene expression (Fig. 4C) was unaffected when compared with animals fed a high-fat diet not preceded by DHA supplementation. However, *Elovl2^−/−^* mice fed a DHA-enriched diet gained body weight (Fig. 4E) and accumulated fat (Fig. 4F) to the same extent as wild-type animals, implying an important role for ELOVL2 and endogenous DHA synthesis in the control of fat storage and energy homeostasis in mammals.

The DHA pretreatment did not result in a fatty liver phenotype, presumably because the exposure time to the high-fat diet was too short to develop a steatotic condition. However, as previous data from our laboratory showed that overexpression of *Elovl2* in adipocytes promoted TG formation and lipid accumulation accompanied by an induction of PPARγ target genes (28), we analyzed hepatic expression of PPARγ and some of its target genes.

We could not detect any differences between *Elovl2^−/−^* mice and wild-type littermates fed on chow diet. However, under high-fat conditions, there was a significant reduction in PPARγ mRNA levels, as well as in PCK1 in the *Elovl2^−/−^* mice, which after DHA supplementation regained similar expression levels as the wild-type mice (Fig. 4G, H). This implies that omega-3 fatty acids, in addition to affecting lipogenesis through SREBP-1c activation, can also modulate lipid homeostasis via an SREBP-1c-independent pathway affecting lipid storage and possibly involving PPARγ and pyruvate cycling.

## DISCUSSION

By generating *Elovl2*-deficient mice, we have recently shown that the ELOVL2 enzyme is required for the elongation of PUFAs of the omega-6 family in testis into C26 PUFAs, which are essential precursors for further elongation by ELOVL4 into C28–C30 carbon atoms that are indispensable for normal sperm formation and fertility in mice (16). As liver lacks ELOVL4, we can show in the present study that the primary function of hepatic ELOVL2 is elongation of 22- into 24-carbon PUFAs for the formation of DPAn-6 and in particular the omega-3 PUFA DHA. Furthermore, as liver is one of the tissues with the highest *Elovl2* expression measured, a 90% reduction in serum levels of DHA in *Elovl2^−/−^* mice indicates that liver is the major contributor of circulating DHA under standard dietary conditions. The data also imply that the ubiquitously expressed elongase ELOVL5, in cooperation with the desaturases FADS1 and 2, are the major enzymes involved in the formation of PUFAs up to 22 carbons in vivo.

Former studies on animal models deficient in the PUFA synthetic pathway by ablation of ELOVL5 (25) or FADS2 (29, 30) also reported reduced levels of DPAn-6 and DHA. However, as these two enzymes, directly and indirectly, control elongation beyond C18, deletion of both *Elovl5* and *Fads2* consequently led to decreased amounts of C20 PUFAs, such as AA and EPA, the levels of which were substantially augmented in the ELOVL2-deficient animals. Interestingly, the *Fads2^−/−^* animals in the two different laboratories display two distinct phenotypes. The animal model obtained by Stroud et al. (30) was characterized by intestinal ulcers, as well as dermatitis that could be prevented by dietary supplementation of AA, which may also explain the absence of this phenotype in our animal model. In contrast, Stoffel et al. (29) did not report either the previously mentioned dermatitis and ulcerations or dysfunction in lipid and carbohydrate metabolism of these animals, which could be due to, for example, differences in the dietary content of AA in the two studies.

Ablation of *Elovl5* revealed a relationship between AA and DHA deficiency in liver and upregulation of lipogenesis via an SREBP-1c-dependent pathway that eventually caused hepatic steatosis in the *Elovl5^−/−^* mice. In contrast, the *Elovl2^−/−^* mice, despite greatly reduced DHA levels and increased hepatic SREBP-1c, did not develop fatty liver but instead exhibited an interesting lean phenotype that was not overcome by high-fat diet treatment.

A relevant explanation for the discrepancy in lipid metabolism between the KO strains is the inverted levels of substrates and products in the *Elovl2^−/−^* mice compared with the other strains. The level of C20 PUFAs, especially EPA, and C22 DPAn-3 was significantly increased in the *Elovl2^−/−^* mice compared with both wild-type littermates and the *Elovl5^−/−^* mice. EPA, as well as DHA, has been shown to be a potent component in prevention and improvement of metabolic disorders, including insulin resistance and hepatic steatosis (3, 4, 31, 32). Whether it is EPA itself or the EPA/DHA ratio that is the major determinant behind the phenomenon seen in the *Elovl2^−/−^* mice is unclear.

Interestingly, in addition to the importance of *Elovl2* in endogenous DHA synthesis, our data on dietary supplementation of DHA in *Elovl2^−/−^* mice also provide new insight into the physiological role of dietary PUFA. Earlier studies emphasizing a beneficial role of diet supplementation with DHA indicate hepatic SREBP-1c as the major target in the control of hepatic lipogenesis and development of fatty liver (33, 34).

In the present study, we can show that disruption of the formation of endogenous DHA positively affects hepatic nSREBP-1c abundance. However, this effect could not be reversed by a 2 week treatment with dietary DHA. Moreover, lack of *Elovl2* products prevented lipid storage in response to a high-fat diet stimulus, whereas restoration of DHA levels in liver and serum of *Elovl2^−/−^* animals, as a result of dietary supplementation, improved the gain of body fat mass. Combined with previous results in our laboratory on *Elovl2* overexpression in fat cells, the present data imply that omega-3 fatty acids, in addition to affecting lipogenesis through SREBP-1c activation, can also modulate lipid homeostasis via an SREBP-1c-independent pathway affecting lipid storage possibly involving PPARγ, pyruvate cycling, and glyceroneogenesis (28, 35, 36). However, more detailed studies are needed to confirm this suggestion.

Our results regarding the downregulation of key lipogenic genes such as *Scd1* and *Fas* in response to high-fat diet, despite increased levels of nSREBP-1c, has, to our knowledge, not been reported previously. However, without overlooking the impact of the fatty acid composition of the diet, the expression levels seems to correlate with the activity of the FAS and SCD1 enzymes, as C16, C16:1, and C18:1 fatty acids are less abundant in liver and serum of animals fed high-fat diet compared with animals maintained on standard chow diet (supplementary Tables III–V). This would imply that SCD1 and FAS enzymes can be regulated in an SREBP-1c-independent manner under certain dietary conditions. Previous reports on SCD1 revealed involvement of this enzyme in the formation of adipose tissue and development of fatty liver due to overnutrition (37, 38). It has been proposed that high-fat diet treatment can lower SCD1 expression most likely via the action of leptin (39), and this effect is not dependent on insulin or the presence of SREBP-1c. In our study, despite downregulation of hepatic lipogenic genes, wild-type animals on high-fat diet showed high levels of hepatic TGs (Fig. 3G), and the mice developed hepatic steatosis (Fig. 3F). This is in accordance with the findings by Ntambi and coworkers (40) that showed that liver-specific *Scd1^−/−^* mice were not protected from obesity induced by high-fat diet, although they were resistant to carbohydrate-induced adiposity. Therefore, one may conclude that hepatic SCD1 enzyme does not play a role in the response to high-fat diet treatment in *Elovl2^−/−^* mice.

We have highlighted here the important role of the ELOVL2 enzyme in the formation of DHA as well as in fat accumulation in hepatocytes during nonalcoholic liver diseases. The impact of ELOVL2 on this is multifactorial, including fatty acid synthesis, storage, and oxidation. The data also show that impaired fat mobilization due to diminished endogenous DHA synthesis can, to a certain extent, be complemented by dietary supplementation. Finally, relatively recent studies suggest that impaired *Elovl2* expression is highly connected with disturbed lipid metabolism and metabolic syndrome in humans (41, 42). Accordingly, *Elovl2* activity represents a therapeutic target for the treatment of DHA deficiency in mammals.

## Supplementary Material

Supplemental Data
