# Supplementary material for: Elovl2 ablation demonstrates that systemic DHA is endogenously produced and is essential for lipid homeostasis in mice
Source: J Lipid Res. 2014 Apr;55(4):718–28. doi: 10.1194/jlr.M046151 (PMC3966705; doi:10.1194/jlr.M046151)
Supplement: Supplemental Data [file supp_55_4_718__index.html]

Elovl2-ablation demonstrate that systemic DHA is endogenously produced and is essential for lipid homeostasis in mice — Elovl2 ablation demonstrates that systemic DHA is endogenously produced and is essential for lipid homeostasis in mice — Supplemental Data 

# Elovl2 ablation demonstrates that systemic DHA is endogenously produced and is essential for lipid homeostasis in mice

## Supplemental Data

**Files in this Data Supplement:**

- Supplemental table 1 - Characterization of Elovl2 -/- mice
- Supplemental table 2 - Fatty acid composition of diets
- Supplemental table 3 - Fatty acid composition of phospholipid pool
- Supplemental table 4 - Fatty acid composition of triglyceride pool
- Supplemental table 5 - Fatty acid composition of serum
- Supplemental table 6 - Fatty acid composition of liver
- Supplemental table 7 - Fatty acid composition of serum
- Supplemental table 8 - qPCR primer sequences
- Supplemental Figure 1 - Overview of experimental setup
