# Supplementary material for: Elovl2 ablation demonstrates that systemic DHA is endogenously produced and is essential for lipid homeostasis in mice
Source: J Lipid Res. 2014 Apr;55(4):718–28. doi: 10.1194/jlr.M046151 (PMC3966705; doi:10.1194/jlr.M046151)
Supplement: Supplemental Data [file supp_M046151_jlr.M046151-1.pdf]

Table SI.

	wild-type	Elovl2 -/-
Body Weight (g)	25.93 ± 0.45	27.01 ± 0.79
Lean Mass (g)	20.55 ± 0.37	21.39 ± 0.62
Fat Mass (g)	2.65 ± 0.17	2.86 ± 0.19
Energy Consumption chow diet (kJ/week)	376.25 ± 7.29	369.23 ± 5.59

Table SI. **Characterization of Elovl2 -/- mice.** Eleven to seventeen week old male Elovl2 -/- and their wild-type littermates were fed standard chow diet. Lean and fat mass were measured using an MRI technique. Body weight was measured weekly. For calculation of energy consumption animals were housed individually and each week food intake was measured.
Data are presented as means ± SEM of 7 mice.
