# Supplementary material for: Elovl2 ablation demonstrates that systemic DHA is endogenously produced and is essential for lipid homeostasis in mice
Source: J Lipid Res. 2014 Apr;55(4):718–28. doi: 10.1194/jlr.M046151 (PMC3966705; doi:10.1194/jlr.M046151)
Supplement: Supplemental Data [file supp_M046151_jlr.M046151-2.pdf]

Table SII.

Fatty acid (% of total)	DHA-enriched diet	chow diet (R70)	high fat diet (D12451)
C12:0	0	0	0
C14:0	0	0.7	1.3
C16:0	4.4	19.0	19.1
C16:1	0	0	1.8
C18:0	2.1	3.5	11
C18:1n-9	30.5	23.0	35.6
C18:2n-6	34.7	49.4	27.2
C18:3n-3	3.5	4.4	2.3
C20:1	0	0	0.7
C20:2	0	0	0.7
C20:4n-6	0	0	0.2
C20:5n-3	5.3	0	0
C22:6n-3	19.5	0	0

Table SII. **Fatty acid composition of diets.**
