# Supplementary material for: Elovl2 ablation demonstrates that systemic DHA is endogenously produced and is essential for lipid homeostasis in mice
Source: J Lipid Res. 2014 Apr;55(4):718–28. doi: 10.1194/jlr.M046151 (PMC3966705; doi:10.1194/jlr.M046151)
Supplement: Supplemental Data [file supp_M046151_jlr.M046151-3.pdf]

Table SIII.

	chow diet		high fat diet	
Fatty acid (mole%)	wild-type	Elovl2-/-	wild-type	Elovl2-/-
C14:0	0.3 ± 0.0	0.3 ± 0.0	0.1 ± 0.0	0.3 ± 0.0*
C16:0	26.6 ± 0.9	22.9 ± 0.9*	20.1 ± 0.8	18.5 ± 0.8
C16:1	1.7 ± 0.1	1.8 ± 0.1	0.6 ± 0.1	0.6 ± 0.1
C18:0	12.9 ± 0.4	13.4 ± 0.6	20.2 ± 0.3	20.4 ± 0.6
C18:1	14.5 ± 0.4	18.2 ± 0.8**	9.7 ± 0.3	11.4 ± 0.3**
C18:2	16.2 ± 0.2	15.0 ± 0.4*	16.8 ± 0.6	16.3 ± 0.6
C18:3n6	0.4 ± 0.0	0.4 ± 0.0	0.4 ± 0.0	0.5 ± 0.0
C18:3n3	0.1 ± 0.0	0.1 ± 0.0	0.1 ± 0.0	0.1 ± 0.0
C20:0	0.4 ± 0.0	0.2 ± 0.1	0.6 ± 0.1	0.6 ± 0.1
C20:1	0.3 ± 0.0	0.5 ± 0.0	0.5 ± 0.1	0.5 ± 0.1
C20:2	0.4 ± 0.0	0.5 ± 0.1	0.5 ± 0.1	0.6 ± 0.1
C20:3n6	1.4 ± 0.1	1.4 ± 0.1	1.1 ± 0.1	0.9 ± 0.1
C20:4n6	14.3 ± 0.6	18.6 ± 0.6***	17.3 ± 0.4	21.5 ± 0.7***
C20:5n3	0.5 ± 0.0	0.8 ± 0.1***	0.3 ± 0.0	0.6 ± 0.0****
C22:0	0.1 ± 0.0	n.d.	0.2 ± 0.1	0.2 ± 0.0
C22:4n6	0.5 ± 0.0	0.9 ± 0.1**	0.4 ± 0.0	0.7 ± 0.0****
C22:5n6	1.0 ± 0.1	0.3 ± 0.0****	0.5 ± 0.0	0.1 ± 0.0****
C22:5n3	0.9 ± 0.1	3.9 ± 0.2****	0.7 ± 0.0	4.1 ± 0.1****
C22:6n3	7.5 ± 0.5	0.8 ± 0.1****	9.8 ± 0.4	2.0 ± 0.1****

Table SIII. **Fatty acid composition of phospholipid pool from liver** of wild-type and *Elovl2 -/-* animals fed standard chow diet or high fat diet for 12 weeks. Values are expressed as mole% and are mean ± SEM of 6 mice.
