# Supplementary material for: Elovl2 ablation demonstrates that systemic DHA is endogenously produced and is essential for lipid homeostasis in mice
Source: J Lipid Res. 2014 Apr;55(4):718–28. doi: 10.1194/jlr.M046151 (PMC3966705; doi:10.1194/jlr.M046151)
Supplement: Supplemental Data [file supp_M046151_jlr.M046151-4.pdf]

Table SIV.

	chow diet		high fat diet	
Fatty acid (mole%)	wild-type	Elovl2-/-	wild-type	Elovl2-/-
C14:0	2.0 ± 0.8	1.6 ± 0.3	0.9 ± 0.1	1.1 ± 0.3
C16:0	33.9 ± 1.7	29.4 ± 1.8	25.1 ± 0.7	25.7 ± 1.1
C16:1	4.9 ± 0.4	5.9 ± 0.3	3.3 ± 0.4	2.7 ± 0.2
C18:0	4.0 ± 0.7	3.0 ± 0.1	3.4 ± 0.3	4.7 ± 0.6
C18:1	31.4 ± 1.5	36.0 ± 2.2	31.7 ± 0.7	31.4 ± 1.9
C18:2	15.2 ± 1.0	14.8 ± 0.3	25.3 ± 0.8	22.2 ± 1.1*
C18:3n6	0.4 ± 0.0	0.4 ± 0.0	0.7 ± 0.1	0.7 ± 0.0
C18:3n3	0.7 ± 0.1	0.6 ± 0.0	1.3 ± 0.1	0.9 ± 0.1***
C20:0	0.5 ± 0.1	0.5 ± 0.1	0.5 ± 0.1	0.7 ± 0.1*
C20:1	0.9 ± 0.2	0.9 ± 0.2	1.2 ± 0.2	1.3 ± 0.1
C20:2	0.5 ± 0.1	0.5 ± 0.1	0.6 ± 0.1	0.7 ± 0.1
C20:3n6	0.4 ± 0.0	0.4 ± 0.0	0.5 ± 0.0	0.6 ± 0.0
C20:4n6	1.7 ± 0.2	2.1 ± 0.2	2.0 ± 0.1	3.1 ± 0.3**
C20:5n3	0.3 ± 0.0	0.5 ± 0.1*	0.4 ± 0.0	0.7 ± 0.0***
C22:0	0.2 ± 0.1	0.1 ± 0.1	n.d.	0.1 ± 0.0
C22:4n6	0.7 ± 0.1	0.9 ± 0.2	0.7 ± 0.1	0.9 ± 0.2
C22:5n6	0.5 ± 0.1	0.2 ± 0.1*	0.4 ± 0.1	0.1 ± 0.0**
C22:5n3	0.4 ± 0.1	1.2 ± 0.1***	0.6 ± 0.1	1.8 ± 0.2***
C22:6n3	1.3 ± 0.1	0.7 ± 0.2	1.3 ± 0.1	0.7 ± 0.2**

Table SIV. **Fatty acid composition of triglyceride pool from liver** of wild-type and *Elovl2 -/-* animals fed standard chow diet or high fat diet for 12 weeks. Values are expressed as mole% and are mean ± SEM of 6 mice.
