# Supplementary material for: Elovl2 ablation demonstrates that systemic DHA is endogenously produced and is essential for lipid homeostasis in mice
Source: J Lipid Res. 2014 Apr;55(4):718–28. doi: 10.1194/jlr.M046151 (PMC3966705; doi:10.1194/jlr.M046151)
Supplement: Supplemental Data [file supp_M046151_jlr.M046151-5.pdf]

Table SV

	chow diet		high fat diet	
Fatty acid (mole%)	wild-type	Elovl2-/-	wild-type	Elovl2-/-
C14:0	1.0 ± 0.1	0.9 ± 0.1	1.0 ± 0.2	0.7 ± 0.0
C16:0	27.7 ± 0.8	23.6 ± 0.5***	20.6 ± 0.3	19.8 ± 0.9
C16:1	2.9 ± 0.1	3.6 ± 0.3	1.4 ± 0.1	1.2 ± 0.1
C18:0	8.3 ± 0.4	9.8 ± 0.6*	15.7 ± 1.1	17.0 ± 0.9
C18:1	18.8 ± 0.7	20.7 ± 1.1	12.9 ± 3.1	13.1 ± 0.5
C18:2	26.9 ± 0.4	23.2 ± 0.7***	31.0 ± 1.1	26.8 ± 0.6*
C18:3n6	0.6 ± 0.0	0.6 ± 0.0	0.5 ± 0.0	0.5 ± 0.0
C18:3n3	0.9 ± 0.1	0.6 ± 0.0***	0.8 ± 0.1	0.5 ± 0.1
C20:0	0.3 ± 0.0	0.2 ± 0.0**	0.3 ± 0.0	0.3 ± 0.0
C20:1	0.8 ± 0.1	0.6 ± 0.0*	0.5 ± 0.0	0.4 ± 0.0
C20:2	0.3 ± 0.0	0.3 ± 0.0	0.5 ± 0.0	0.5 ± 0.0
C20:3n6	0.7 ± 0.0	0.9 ± 0.0*	0.6 ± 0.0	0.6 ± 0.0
C20:4n6	6.7 ± 0.5	11.6 ± 1.1**	10.1 ± 0.7	14.6 ± 0.8**
C20:5n3	0.5 ± 0.1	0.7 ± 0.0	0.3 ± 0.0	0.6 ± 0.0**
C22:0	0.1 ± 0.0	0.1 ± 0.0	0.1 ± 0.0	0.1 ± 0.0
C22:4n6	0.3 ± 0.0	0.5 ± 0.1**	0.2 ± 0.0	0.4 ± 0.0**
C22:5n6	0.4 ± 0.1	0.1 ± 0.0**	0.2 ± 0.0	0.2 ± 0.0
C22:5n3	0.4 ± 0.0	1.7 ± 0.1****	0.4 ± 0.0	1.8 ± 0.1****
C22:6n3	2.3 ± 0.3	0.4 ± 0.1****	2.9 ± 0.3	0.9 ± 0.1***

Table SV. **Fatty acid composition of serum** from wild-type and *Elovl2 -/-* animals fed standard chow diet or high fat diet for 12 weeks. Values are expressed as mole% and are mean ± SEM of 6 mice.
