# Supplementary material for: Elovl2 ablation demonstrates that systemic DHA is endogenously produced and is essential for lipid homeostasis in mice
Source: J Lipid Res. 2014 Apr;55(4):718–28. doi: 10.1194/jlr.M046151 (PMC3966705; doi:10.1194/jlr.M046151)
Supplement: Supplemental Data [file supp_M046151_jlr.M046151-6.pdf]

Table SVI.

	chow/high fat diet		DHA/high fat diet	
Fatty acid (mole%)	wild-type	Elovl2-/-	wild-type	Elovl2-/-
C14:0	0.7 ± 0.0	0.5 ± 0.0	0.7 ± 0.0	0.6 ± 0.1
C16:0	27.6 ± 0.5	23.2 ± 0.7	27.9 ± 0.7	26.7 ± 1.1
C16:1	2.0 ± 0.1	1.6 ± 0.1	2.0 ± 0.1	2.0 ± 0.3
C18:0	11.3 ± 0.6	13.4 ± 0.7	9.3 ± 0.5	11.5 ± 1.4
C18:1	19.1 ± 0.4	20.0 ± 0.5	21.5 ± 0.2	19.5 ± 0.9
C18:2	23.8 ± 0.6	23.1 ± 0.3	24.5 ± 0.5	24.3 ± 0.9
C18:3n6	0.7 ± 0.0	0.6 ± 0.0	0.6 ± 0.0	0.5 ± 0.0
C18:3n3	0.9 ± 0.1	0.6 ± 0.0	0.9 ± 0.0	0.8 ± 0.1
C20:0	0.7 ± 0.1	0.6 ± 0.0	0.6 ± 0.0	0.5 ± 0.1
C20:1	0.4 ± 0.1	0.6 ± 0.1	0.4 ± 0.1	0.5 ± 0.1
C20:2	0.5 ± 0.0	0.6 ± 0.1	0.5 ± 0.0	0.5 ± 0.0
C20:3n6	0.5 ± 0.0	0.5 ± 0.1	0.4 ± 0.0	0.5 ± 0.0
C20:4n6	7.8 ± 0.5	11.5 ± 0.6	6.0 ± 0.5	7.6 ± 1.0
C20:5n3	0.1 ± 0.0	0.5 ± 0.0	0.3 ± 0.0	0.7 ± 0.1
C22:4n6	0.3 ± 0.0	0.6 ± 0.0	0.3 ± 0.0	0.2 ± 0.0
C22:5n6	0.3 ± 0.0	n.d.	0.2 ± 0.0	n.d.
C22:5n3	0.3 ± 0.0	1.5 ± 0.1	0.3 ± 0.0	0.7 ± 0.0
C22:6n3	3.1 ± 0.2	0.5 ± 0.0	3.4 ± 0.2	2.7 ± 0.2

Table SVI. **Fatty acid composition of liver** from wild-type and *Elovl2 -/-* animals fed standard chow diet, followed by 2 weeks of high fat diet (Chow/HF) or pre-fed for 2 weeks DHA-enriched diet, followed by 2 weeks of high fat diet (DHA/HF). For the experimental overview see Fig. SI. Values are expressed as mole% and are mean ± SEM of 6 mice.
