# Supplementary material for: Elovl2 ablation demonstrates that systemic DHA is endogenously produced and is essential for lipid homeostasis in mice
Source: J Lipid Res. 2014 Apr;55(4):718–28. doi: 10.1194/jlr.M046151 (PMC3966705; doi:10.1194/jlr.M046151)
Supplement: Supplemental Data [file supp_M046151_jlr.M046151-7.pdf]

Table SVII.

	chow/high fat diet		DHA/high fat diet	
Fatty acid (mole%)	wild-type	Elovl2-/-	wild-type	Elovl2-/-
C14:0	0.6 ± 0.1	0.9 ± 0.4	0.8 ± 0.1	0.9 ± 0.4
C16:0	16.6 ± 0.8	15.5 ± 0.5	16.1 ± 0.3	16.8 ± 0.9
C16:1	0.9 ± 0.0	0.8 ± 0.1	0.8 ± 0.0	0.9 ± 0.1
C18:0	16.6 ± 0.6	16.3 ± 1.0	15.8 ± 0.7	16.5 ± 0.8
C18:1	11.9 ± 1.0	12.9 ± 2.1	11.1 ± 0.3	12.0 ± 0.4
C18:2	27.8 ± 0.6	27.7 ± 0.6	28.0 ± 0.4	28.0 ± 0.9
C18:3n6	0.5 ± 0.1	0.5 ± 0.0	0.4 ± 0.1	0.3 ± 0.0
C18:3n3	0.5 ± 0.1	0.5 ± 0.1	0.5 ± 0.0	0.5 ± 0.1
C20:0	0.6 ± 0.0	0.8 ± 0.1	1.0 ± 0.0	0.8 ± 0.1
C20:1	0.4 ± 0.1	0.5 ± 0.1	0.4 ± 0.0	0.4 ± 0.1
C20:2	0.6 ± 0.1	0.5 ± 0.1	0.5 ± 0.0	0.4 ± 0.1
C20:3n6	0.7 ± 0.0	0.6 ± 0.0	0.8 ± 0.0	0.7 ± 0.0
C20:4n6	14.9 ± 0.7	17.3 ± 1.7	14.6 ± 0.8	14.8 ± 1.6
C20:5n3	0.3 ± 0.0	0.6 ± 0.1	0.5 ± 0.0	1.2 ± 0.3
C22:0	0.7 ± 0.1	0.7 ± 0.1	0.9 ± 0.0	0.9 ± 0.1
C22:4n6	0.4 ± 0.0	0.7 ± 0.1	0.3 ± 0.0	0.4 ± 0.1
C22:5n6	0.4 ± 0.0	0.1 ± 0.0	0.2 ± 0.0	0.1 ± 0.1
C22:5n3	0.5 ± 0.1	2.2 ± 0.3	0.5 ± 0.1	1.0 ± 0.1
C22:6n3	5.2 ± 0.4	0.8 ± 0.2	6.7 ± 0.4	3.4 ± 0.9

Table SVII. **Fatty acid composition of serum** from wild-type and *Elovl2 -/-* animals fed standard chow diet, followed by 2 weeks of high fat diet (Chow/HF) or pre-fed for 2 weeks DHA-enriched diet, followed by 2 weeks of high fat diet (DHA/HF). For the experimental overview see Fig. SI. Values are expressed as mole% and are mean ± SEM of 6 mice.
