# Supplementary material for: Elovl2 ablation demonstrates that systemic DHA is endogenously produced and is essential for lipid homeostasis in mice
Source: J Lipid Res. 2014 Apr;55(4):718–28. doi: 10.1194/jlr.M046151 (PMC3966705; doi:10.1194/jlr.M046151)
Supplement: Supplemental Data [file supp_M046151_jlr.M046151-8.pdf]

Table SVIII. qPCR primer sequences for mRNA measurements

Gene	Forward	Reverse
18S	AGTCCCTGCCCTTTGTACACA	CGATCCGAGGGCCTCACTA
SREBP1c	GGAGCCATGGATTGCACATT	GGCCCGGGAAGTCACTGT
FAS	GCTGCCCAAACTTCAGGAAAT	AGAGACGTGTCACTCCTGGACTT
SCD1	TTCTTCTCTCACGTGGGTTG	CGGGCTTGTAGTACCTCCTC
PPARgamma	GAAAGACAACGGACAAATCACC	GGGGGTGATATTTTGAACTTG
Pck1	ACATTGCCTGGATGAAGTTTG	GGCATTTGGATTTGTCTTCAC
