# Supplementary figures and images for: Elovl2 ablation demonstrates that systemic DHA is endogenously produced and is essential for lipid homeostasis in mice
Source: J Lipid Res. 2014 Apr;55(4):718–28. doi: 10.1194/jlr.M046151 (PMC3966705; doi:10.1194/jlr.M046151)

Fig.SI. Overview of experimental setup

DHA supplementation experiment

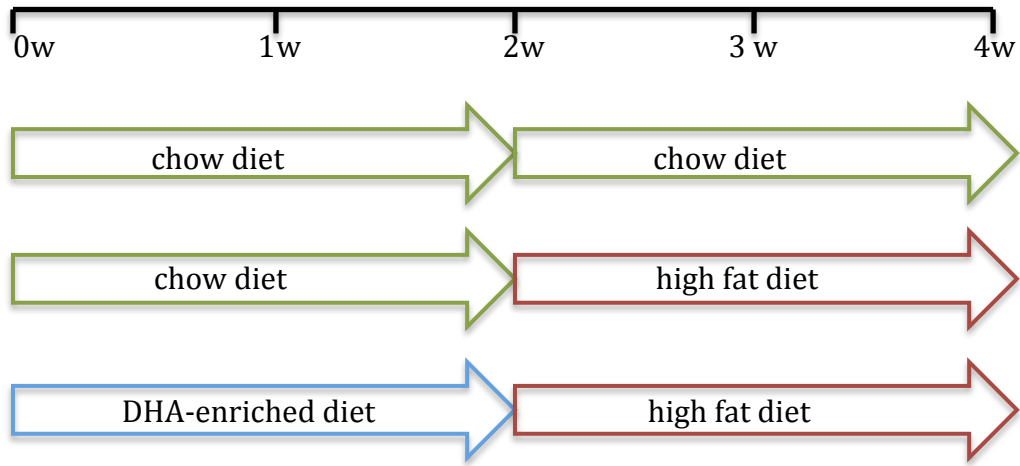

Supplement: Supplemental Data [file supp_M046151_jlr.M046151-9.pdf]
